# Which factors are associated with the prevalence of meniscal repair?

**DOI:** 10.1186/s12891-021-04107-w

**Published:** 2021-03-22

**Authors:** Xiaoxiao Song, Dongyang Chen, Xinsheng Qi, Qing Jiang, Caiwei Xia

**Affiliations:** 1 Department of Orthopedics, Affiliated Taikang Xianlin Drum Tower Hospital, Medical school of Nanjing University, Nanjing, Jiangsu P.R. China; 2 Department of Sports Medicine and Adult Reconstructive Surgery, Nanjing Drum Tower Hospital, School of Medicine, Nanjing University, 321 Zhongshan Road, Nanjing, 210008 Jiangsu PR China

**Keywords:** meniscal repair, meniscectomy, concurrent ACL injury, age, duration of injury

## Abstract

**Abstract:**

Purpose To investigate the potential factors associated with the prevalence of meniscal repair

Methods Patients who received partial meniscectomy or meniscal repair in our institution from Jan 2015 to Dec 2019 were included in current study. The inclusion criteria were (1) meniscus tear treated using meniscectomy or repair, (2) with or without concomitant anterior cruciate ligament reconstruction, (3) not multiligamentous injury. Demographic data, including sex, age, body mass index (BMI), injury-to-surgery interval and intra-articular factors such as the location of injury, medial or lateral, ACL rupture or not and the option of procedure (partial meniscectomy or repair) were documented from medical records. Univariate analysis consisted of chi-square. Multivariate logistic regression was then performed to adjust for confounding factors.

Results 592 patients including 399 males and 193 females with a mean age of 28.7 years (range from 10 to 75 years) were included in current study. In the univariate analysis, male (*p* = 0.002), patients aged 40 years or younger (*p* < 0.001), increased weight (*p* = 0.010), Posterior meniscus torn (0.011), concurrent ACL ruputure (*p* < 0.001), lateral meniscus (*p* = 0.039) and early surgery (*p* < 0.001) were all associated with the prevalence of meniscal repair. However, After adjusting for confounding factors, we found that age (OR, 0.35; 95% CI, 0.17 - 0.68, *p* = 0.002), ACL injury (OR, 3.76; 95% CI, 1.97 – 7.21, *p* < 0.001), side of menisci (OR, 3.29; 95% CI, 1.43 – 7.55, *p* = 0.005), site of tear (OR, 0.15; 95% CI, 0.07 – 0.32, *p* < 0.001), and duration of injury (OR, 0.46; 95% CI, 0.28 – 0.82, *p* = 0.008) were associated with the prevalence of meniscus repair.

Conclusions Meniscal tear in aged patients especially those with concomitant ACL injury is likely to be repaired. Additionally, in order to increase the prevalence of repair and slow down progression of OA, the surgical procedure should be performed within two weeks after meniscus tear especially when the tear is located at lateral meniscal posterior.

**Study design:**

Case-control study; level of evidence, 3.

## Introduction

Meniscus is a vital intra-articular structure to maintain knee stability and to slow down the progression to osteoarthritis (OA) [1, 2]. It also play an important role in load transmission, shock absorption and joint lubrication [3, 4]. With the development of diagnostic techniques for meniscal injury, the volume of arthroscopic procedures to treat injuried meniscus dramatically increased in recent years. Partial meniscectomy had been widely used for patients who underwent a failure of nonoperative treatment in early years [5, 6]. However, with deep understanding of the biomechanical function of meniscus, surgeon realized that meniscectomy surgery could relieve pain and improve knee function at short-term follow up, but the loss of meniscal tissue also accelerate the development of early OA in the future [7, 8]. In comparison with partial meniscectomy, meniscus repair procedure can restore its biomechanical function and prevent the development of knee OA by preserving meniscal tissue at long-term follow up. In the above setting, a consensus had been reached that meniscal tissue should be preserved as much as possible [9–11].

The surgeon would determine whether to repair the injuried meniscus for patients based on the following two criterions: the location of meniscal tear and the severity of meniscal injury. If the meniscal tear located in red-red zone or the remaining meniscal tissue is adequate, the repair procedure may be performed [12]. However we cannot give them an accurate answer, when patients consult about the surgical plan before operation as the severity and the vascularity of meniscus are subjective standards and was able to be precisely evaluated based on surgeon’s experience under the arthroscope [13, 14] . To our knowledge, studies tried to find factors associated with the prevalence of meniscus repair were rare. Thus, we performed this study to investigate the potential factors associated with the choice of repair procedure and to make the standards for repair surgery more specific. So that we can counsel patients about the options and expectations preoperatively. Additionally, identifying these factors can increase the prevalence of patients undergoing meniscal repair. We hypothesized that young patients with meniscal posterior horn tear are more likely to underwent repair surgery.

## Method

Institutional review board approval was waived as no patients’ private information was involved. 592 patients who received partial meniscectomy or repair in our institution from Jan 2015 to Dec 2019 by three high experienced surgeons were included in current study. The inclusion criteria were (1) meniscus tear treated using meniscectomy or repair, (2) with or without concomitant anterior cruciate ligament reconstruction, (3) not multiligamentous injury. Demographic data, including sex, age, body mass index (BMI), injury-to-surgery interval and intra-articular factors such as the location of injury, medial or lateral, ACL rupture or not and the option of procedure (partial meniscectomy or repair) were documented from medical records. Age was divided into older group (>40) and younger group (≤40), the duration from injury to surgery was defined as delayed group (>2weeks) and early group (≤2weeks), BMI was divided into three groups (≤24Kg/m^2^,24-27 Kg/m^2^,and ≥27 Kg/m^2^ ),.

The meniscal injury was determined by clinical examination, magnetic resonance imaging (MRI) and intraoperative findings. Surgical procedure were performed by three experienced surgeons (being numbered as 1, 2 and 3, respectively) and the procedure was uniform. Anterolateral and anteromedial knee portals were utilized for all patients. Under arthroscopy, the meniscus tear patterns, the site and the potential vascularity were directly seen by the surgeons. Then the surgeon would determine a suitable procedure for each patient based on the following standards: (1) the location of meniscal tear; (2) the severity of meniscal injury. Meniscal repair was implemented through all-inside technique using meniscal repair device (Fast-Fix; Smith & Nephew) for all patients with meniscal posterior, body or anterior tear. According to the length of tear, one to seven sutures were needed. When repair was not viable, injuried meniscus will be trimmed until a stable peripheral rim was achieved. In the case of a concurrent ACL injury, the ACL reconstruction will be performed firstly by using peroneal longus tendon as graft.

### Statistical analysis

Statistical analyses were achieved by using the SPSS, version 23.0 (SPSS Inc., Chicago, IL, USA). To assess association between demographic data and the prevalence of partial meniscectomy procedure, chi-squared test were used. In the multivariate analysis, binary regression were used to identify the independent variables for meniscal partial meniscectomy. A p value less than 0.05 were considered statistical significant.

## Results

**Fig. 1:**
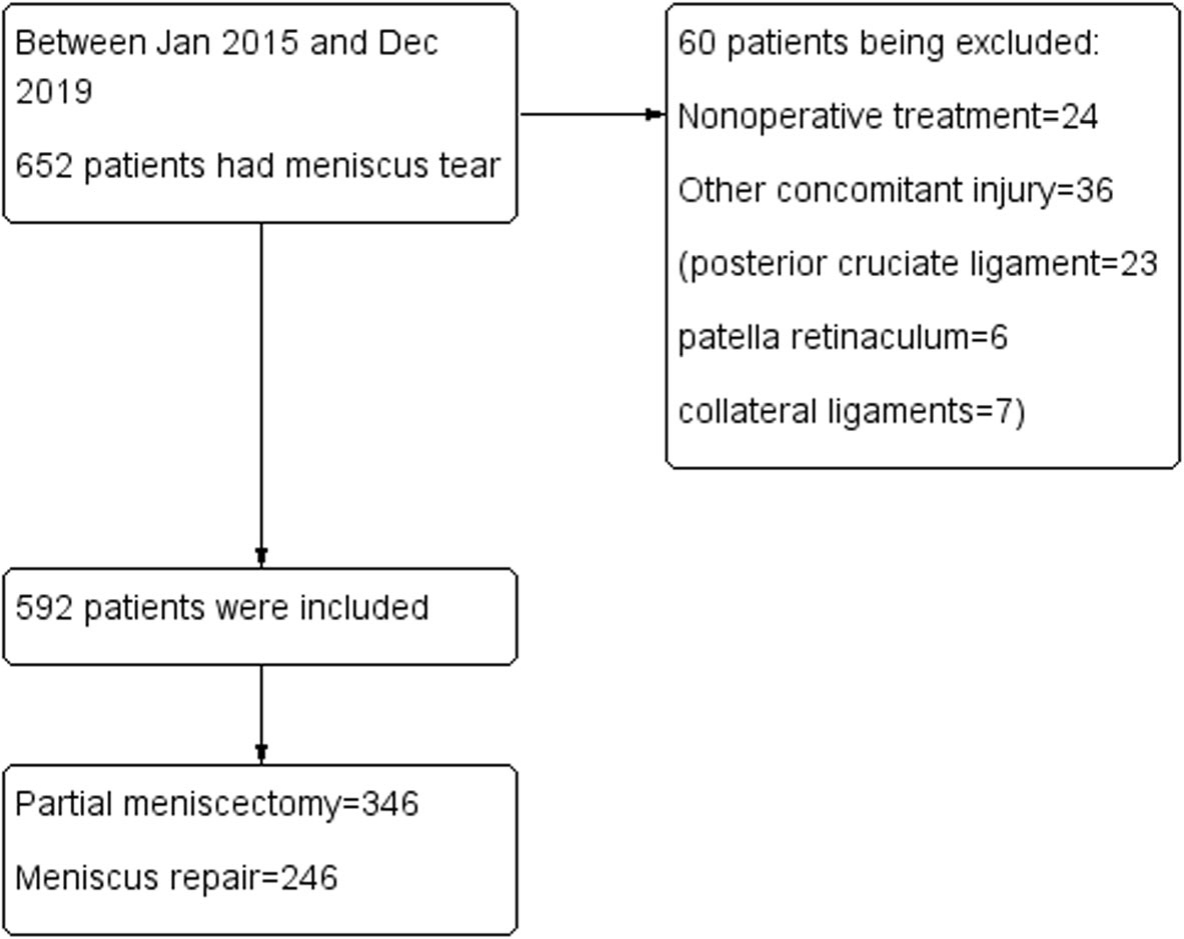
Flowchart of included participants

From Jan 2015 to Nov 2019, 592 patients including 399 males and 193 females with a mean age of 28.7 years (range from 10 to 75 years) were included in current study. Of these patients, 368 patients underwent concomitant ACL reconstruction, 224 patients underwent isolated meniscal surgery. Partial meniscectomy was performed on 346 patients, the remaining 246 patients received meniscus repair operation (Fig. 1). Those demographic baseline data were shown in Table 1.

**Table 1:** Demographic baseline data

	Partial meniscectomy (%)	Repair (%)	*P*-value
Gender			0.002
male	216 (54.1)	183 (45.9)	
female	130 (67.4)	63 (32.6)	
Age			<0.001
≤40	213 (50.1)	212 (49.9)	
>40	133 (79.6)	34 (20.4)	
Weight			0.010
≤60	75 (66.4)	38 (33.6)	
60-90	246 (58.3)	176 (41.7)	
≥90	20 (40.8)	29 (59.2)	
BMI			0.544
≤24	170 (60.3)	112 (39.7)	
24-27	104 (58.4)	74 (41.6)	
≥27	67 (54.5)	56 (45.5)	
Site of tear			0.011
Multiple	40 (55.6)	32 (44.4)	
Anterior	23 (76.7)	7 (23.3)	
Body	43 (69.4)	19 (30.6)	
Posterior	114 (51.8)	106 (48.2)	
ACL injury			<0.001
Yes	160 (43.5)	208 (56.5)	
No	186 (83.0)	38 (17.0)	
Duration of injury			<0.001
≤2weeks	59 (39.3)	91 (60.7)	
>2weeks	287 (60.9)	155 (35.1)	
Laterality of meniscus			0.039
Lateral	170 (58.8)	119 (41.2)	
Medial	132 (62.9)	78 (37.1)	
Both	44 (47.3)	49 (52.7)	
Surgeons			0.211
1	141 (58.5)	100 (41.5)	
2	75 (57.3)	56 (42.7)	
3	130 (59.1)	90 (40.9)	

In the univariate analysis, seven variables were found to be associated with the prevalence of meniscus repair. male patients (*p* = 0.002), younger than 40 years (*p* < 0.001), increasing of body weight (*p* = 0.010), Posterior meniscus torn and multiple site torn injury (*p* = 0.011), with concurrent ACL ruputure (*p* < 0.001), performing surgery within two weeks after injury (*p* < 0.001), and lateral or both sides injury (*p* = 0.039). Detailed data are displayed in Table 1.

Then, these variables were analyzed in multivariate logistic regression model. After adjusting for confounding factors, we found that age (OR, 0.35; 95% CI, 0.17 - 0.68, *p* = 0.002), ACL injury (OR, 3.76; 95% CI, 1.97 – 7.21, *p* < 0.001), side of menisci (OR, 3.29; 95% CI, 1.43 – 7.55, *p* = 0.005), site of tear (OR, 0.15; 95% CI, 0.07 – 0.32, *p* < 0.001), and duration of injury (OR, 0.46; 95% CI, 0.28 – 0.82, *p* = 0.008) were associated with the prevalence of meniscus repair (Table 2).

**Table 2:** Multivariable analysis of factors associated with the prevalence of meniscal repair

	Regression coefficient	95%CI	*P*-value
Gender	1.14	0.67 to 1.96	ns
Age	0.35	0.17 to 0.68	0.002
Weight	1.03	0.55 to 1.93	ns
Site of tear	0.15	0.07 to 0.32	<0.001
ACL injury	3.76	1.97 to 7.21	<0.001
Duration of injury	0.46	0.28 to 0.82	0.008
Laterality of meniscus	3.29	1.43 to 7.55	0.005

## Discussion

The most important findings of current study were that younger age, early arthroscopic surgery (<2 weeks), lateral meniscus tear, posterior horn tear and accompanying with ACL injury were associated with the prevalence of meniscal repair surgery.

The clinical outcomes between partial meniscectomy and meniscal repair has been hotly debated in recent years. In a study performed by Kyu et al [15], they observed the difference of patients-reported outcomes among patients who had underwent meniscectomy or repair for at least 10 years follow-up and claimed that meniscal repair had a superior clinical outcomes. Stein et al [16] evaluated the rate of return to sports in athletes and found that patients underwent meniscal repair had a higher rate of return to sports (96.15%) comparing with those underwent partial meniscectomy (50%) at 8.8-year follow-up. While the re-operation rates of meniscal repair was likely to be higher than that of meniscectomy at short-term follow-up [17]. Neverthless, given the increased risk of partial meniscectomy predisposing patients to early onset degenerative changes, preversation of meniscal tissue should be attempted whenever possible [9–11]. However, to our knowledge, no study was conducted to assess the potential factors associated with the prevalence of meniscal repair. In this setting, we performed this study.

The influence of age on the clinical outcomes after arthroscopic meniscal repair have been widely reported in previous literature. Mike et al [18] found that younger age could improve the knee function and enhanced the healing rate of repaired meniscus significantly which was consistent with another study [19]. However, substantial articles also shown an opposite conclusions. In a respective cohort study with 16-years follow-up, Steadman et al [20] found that the failure rate of meniscus repair, knee function and patient satisfaction were not significant in patients who are 40 years or younger and older than 40 years. Their findings were supported by Sarah et al [21] and Shane et al [22]. In current study, surgeons tend to perform meniscus repair for younger patients (≤40 years). Michela et al [23]. found that the meniscal fibrocartilage became disrupt and proteoglycan content increased in those patients with OA (mean age: 72) and concluded that age was the strongest factor determining post-operative outcome. Additionally, the high possibilty of an early onset of OA when meniscal tissue is resected totally or partially also makes surgeons worried.

In our study, 472/512 (84.5%) traumatic meniscus tear were accompanyed by a concurrent ACL rupture which was similar to other articles’ results [24]. We found that ACL injury was the most important factor associated with the prevalence of meniscal repair. Many articles had reported a higher healing rate in patients undergoing a concomitant ACL reconstruction compared with those receiving isolated meniscus surgery [24–26]. They claimed that the intra-articular growth factors in bleeding from tunnels created an ideal enviroment for meniscus healing [27, 28]. Besides, most of these meniscis tear accompanying with ACL injury were sports-related which was more common among younger patients. The above two reasons may be explained for the popularity of meniscus repair in patients having ACL injury concomitnatly.

The association between BMI and the clinical outcomes of meniscal repair had been widely studied in previous literature. In a meta-analysis [29], the authors concluded that the failure rate of repair surgery was not significant difference among patients with high BMI or low BMI. In another study, BMI was found to be inversely correlated with IKDC and Tegner activity level scores. In our study [30], we found that when performing meniscus repair surgery, surgeons may not consider patients’ BMI as a contraindication for this surgery, but high body weight will be. We speculated that the large body weight could result in a high stress on joint and may consequently lead to serious meniscus injury but BMI can be influenced by patients’ height.

Nonoperative treatment was commonly recommended to patients with degenerative meniscal injury. Partial meniscectomy or meniscus repair will be suggested when meniscal symptoms, including pain and knee locking cannot be relieved after a two-week conservative treatment in our institution. Haroon et al [24] found that the failure rate of meniscal repair was lower if meniscus is repaired within 6weeks in those patients acommpanying by ACL injury. However, we found that when arthroscopic surgery was delayed to two weeks later after injury, the ratio of undergoing meniscal repair will be reduced significantly. Meniscus is the second knee stabilizer. The meniscus tear will become more and more serious, when meniscal surgery was postponed especially when ACL deficient [31, 32]. We speculated that the severely damaged meniscus may influence surgeons’ decisions on meniscal repair as its poor healing potential.

As we all know, meniscus are classified as three zone, that is the white-white zone, white-red zone and red-red zone according to the vascularity [17]. Theoretically, tear within the vascular zone have a higher healing rate comparing with those in avascular zone [17, 24, 29]. In a study performen by Zhu et al [33]. found that human cartilage-derived morphogenetic protein-2 (hCDMP-2) growth factor could promote meniscal fibrocartilage healing by regenerating fibrocartilage-like tissue. The tissue in vascular zone was regenerated more rapidly than that in the avascular zone. In another study [34], the authors concluded that tear in red-red zone were more appropriate to be repaired as the higher healing rate. Theoretically, the meniscal vascularity is a vital factor that may affect surgeon’s treatment options. However, in our study, we did not evaluate the influence of tear zones on the prevalence of repair as they were not recorded. Some authors believed that the posterior horn root tear should be repaired whenever possible, because detachment of posterior root can disrupt continuity of the circumferential fibers and lead to loss of hoop tension [15, 30]. We also observed that surgeons tend to repair meniscal posterior horn.

No consensus being reached on whether the side of repair have differential influence on the clinical outcomes and the failure rate [29]. Some studies found lateral meniscal repair had a higher success rate [30], some other articles hold an opposite opinion, while much more literature shown that the the failure rate was not associated with the side of repair [29]. In current study, those patients with lateral meniscus tear and both sides injury were more likely to receive meniscal repair. The possible explainations for this finding may be that lateral meniscus injury was easy to repair with “4” character sign and both sides injury mean the tear was serious.

Despite many factors had been studied and shown to be associated with the high failure rate of maniscal repair or partial meniscectomy, whether they can affect surgeons’ decision on treatment options and which will affect have not yet been evaluated. In current study, we found that aged patients especially those with concomitant ACL injury were more likely to receive maniscal repair. Additionally, in order to increase the prevalence of repair and slow down progression of OA, the surgical procedure should be performed within two weeks after meniscus tear. Lateral meniscal posterior horn injury have a higher opportunity for meniscus repair.

However, our study have some limitations. Firstly, tear zone of meniscus should be an important predictive factor for treatment options, but we failed to assess it because of data loss. More study is needed, in the future, to investigate whether meniscal tear within the red-red zone is more likely to be repaired compared with those within white-white zone. Secondly, the data in this study were collected from a single medical center in China. Thus,more studies are needed in the future to investigate the relationship between patient-related factors and the prevalence of meniscal repair. Finally, option bias may exists because three different high experienced surgeons performed all arthroscopic meniscal operation, however, we believed this can reflect the realistic clinical issue.

### Conclusion

Patients should be informed that their meniscus are more likely to be repaired if they are young and operation is performed within 2 weeks after injury, and the meniscus tear is located at lateral meniscal posterior horn especially with concomitant ACL injury.

## Data Availability

The datasets used and/or analyzed during the current study will be available from author XXS on a reasonable request and no material or illustrations have been previously published

## Abbreviations

ACL: Anterior cruciate ligament
BMI: Body mass index
MRI: Magnetic resonance imaging
